# Comparison of sentinel lymph node biopsy guided by indocyanine green, blue dye, and their combination in breast cancer patients: a prospective cohort study

**DOI:** 10.1186/s12957-017-1264-7

**Published:** 2017-11-02

**Authors:** Jiajia Guo, Houpu Yang, Shu Wang, Yingming Cao, Miao Liu, Fei Xie, Peng Liu, Bo Zhou, Fuzhong Tong, Lin Cheng, Hongjun Liu, Siyuan Wang

**Affiliations:** Peking University People’s Hospital Breast Center, NO 11, Xizhimen South Street, Xicheng District, Beijing, 10044 People’s Republic of China

**Keywords:** Breast neoplasm, Indocyanine green, Sentinel lymph node biopsy, Lymphography

## Abstract

**Background:**

Recent studies show that near-infrared (NIR) fluorescence imaging using indocyanine green (ICG) has the potential to improve the performance of sentinel lymph node (SLN) mapping. The current cohort study was designed to assess the value of the combination of ICG and methylene blue (MB) dye in patients undergoing SLN biopsy.

**Methods:**

A prospective self-controlled trial was designed to detect the difference in the detection efficacies of ICG, MB, and combined ICG and MB (ICG + MB) navigation methods. Between 2010 and 2013, 198 consecutive early breast cancer patients eligible for sentinel lymph node biopsy were enrolled and 200 biopsy procedures were performed by injection of both ICG and MB. SLNs were searched and removed under the guidance of fluorescence and/or blue dye. The mapping characteristics, the detection rate of SLNs and positive SLNs, and the number of SLNs of ICG, MB, and ICG + MB were compared. Injection safety of ICG and MB was evaluated.

**Results:**

Fluorescence imaging of lymphatic flow, which is helpful to locate the incision site, could be seen in 184 of 200 procedures. The nodal detection rate of ICG, MB, and ICG + MB samples was 97, 89, and 99.5% (*χ* ^2^ = 26.2, *p* < 0.001), respectively, with the combination method yielding a superior identification result. The addition of ICG to the MB method resulted in the identification of more lymph nodes (median 3 versus 2) and more positive axillas (22.7% involved axillas were discovered by fluorescence only) than either method alone. No acute or chronic allergic reaction was observed in this study. However, 23 patients (23/82) who received breast-conserving therapy reported temporary skin staining, and 5 patients had permanent tattooing. Palpable subcutaneous nodules at the injection sites were reported in nine patients. There were no reports of skin necrosis.

**Conclusions:**

The lymphatic navigation by ICG fluorescence detects SLNs at a high detection rate and improves the mapping performance when added to the MB method. The novel ICG + MB dual tracing modality, without involvement of radioactive isotopes, exhibits great potential as an alternative to traditional standard mapping methods.

**Trial registration:**

ACTRN12612000109808. Retrospectively registered on 23 January 2012.

## Background

Knowledge of the regional lymph node status is essential to establishing staging and prognostic outcomes of breast cancer. Axillary lymph node staging by sentinel lymph node biopsy (SLNB) is a widely used method and is now regarded as a standard of care in patients without clinical evidence of axillary lymph node metastasis in early breast cancer [1].

The existing standard SLNB method is a dual technique involving the injection of a technetium-99m (^99m^Tc)-labeled nanocolloid and blue dye [2]. Despite the reports of effectiveness and good safety data, the involvement of radioisotopes creates logistical challenges, including isotope handling and disposal, staff training, and legislative requirements, as well as the reluctance of patients and personnel to be exposed to radiation. Constraints of radioisotopes have led to the development of non-radioactive alternative methods. Quite a few institutions have attempted to perform SLNB using blue dye alone [3–6]. As an alternative dye tracer, methylene blue (MB) does not have the restrictions of isosulfan blue and patent blue, such as life-threatening anaphylaxis and international shortage. The MB alone method has gained widespread popularity worldwide, especially in developing countries like China [7–11]. However, some investigators maintained that the use of blue dye alone leads to a lower SLN identification rate [12], which might draw concerns about the potential adverse impact on long-term prognosis [13].

SLNB with near-infrared (NIR) fluorescence imaging using indocyanine green (ICG) was recently introduced and was reported to be a highly sensitive method for SLN detection [14–16]. In this method, ICG is injected, and its progress through the lymphatic ducts to SLNs is tracked using an excitation illumination system combined with a high-sensitivity camera that detects the emitted fluorescence. As NIR could be transcutaneously detected similar to radioactive agents, the combination of ICG and MB shows great potential as an alternative to standard dual mapping methods [14, 17–19]. Hence, we designed the present prospective study to evaluate whether combining ICG and MB could improve the SLN tracing performance in early breast cancer patients.

## Methods

### Patients

Between January 2010 and March 2013, a total of 198 consecutive patients (196 with unilateral cancers and 2 with bilateral disease) with early breast cancer, as confirmed by core needle biopsy or open biopsy, and clinically negative axillas were enrolled in the present study. The present study was approved by the Ethical Committee of Peking University People’s Hospital, Beijing and was registered as the Australian and New Zealand Clinical Trials Registry No 12612000109808. Patients with tumors > 5 cm, clinically or radiologically suspicious lymph nodes, inflammatory breast cancer, distant metastatic tumor, previous axillary surgery, or hypersensitivity to iodine or indocyanine green were excluded from the study. Written informed consent was obtained from all patients.

### Procedure

MB (Jizhou Pharmaceutical, Suzhou, China) was diluted to a final concentration of 1% with saline from an original concentration of 2%. The ICG (Dandong Pharmaceutical, Jilin, China) agent used in this study included iodine. Twenty-five milligrams of stock ICG powder was dissolved in 10 ml of distilled water and was then diluted to a final concentration of 0.05%. Immediately before surgery, 1 ml of 1% MB was subdermally injected into the periareolar region. Five minutes later, 1 ml of ICG solution (1.25 mg) was intradermally injected into two to four sites in the same periareolar area region. The movement of the tracer in the periareolar region was facilitated by massage. ICG fluorescence was stimulated and detected by a hand-held fluorescence detector (MingDe Medicine, China, elicitation wavelength of 780 nM and acquisition wavelength of 735 nM). The lymphatic drainage was traced by fluorescence navigation and visualized on a monitor in real time. The fluorescent signal was followed from the injection sites to the axilla (occasionally to the intramammary region), and an incision was made to start the SLNB where the fluorescence disappeared. Fluorescent lymph nodes (ICG-positive) and/or blue nodes (MB-positive) were localized and excised. Then, the axilla was inspected for residual fluorescent or blue nodes. The excised nodes were categorized as fluorescent+/blue+, fluorescent+/blue-, or fluorescent−/blue+. All removed nodes were sent for histological examination following an institutional standard protocol. Patients with positive SLNs underwent axillary clearance, and benign axillas were spared.

Adverse events, including skin lesions, allergic reactions and any other complication considered to be caused by the tracers, were recorded in indicated cases according to patients’ complaint, physical examination, and imaging tests.

### Study design and statistical analysis

The trial was composed to evaluate whether the ICG + MB modality was superior to MB alone in terms of its ability to detect SLNs.

The detection rate was calculated by the number of successful mappings divided by the total number of mapping cases performed for each modality. To estimate the number of samples, we assumed a detection rate of 85% for the control method (dye alone) [20] and set *δ* at 5%. We found that 200 procedures were needed to demonstrate superiority between the two methods with a power of 80% and a significance (*α*) of 5%. The calculation was performed by using PASS 11 software (NCSS, LLC. Kaysville, Utah, USA).

For statistical analysis, SAS software (version 9.2) was used. A chi-square test was used to compare the detection rates. To compare the median number of SLNs between groups, a non-parametric Mann-Whitney *U* test was used.

## Results

### ICG mapping

**Fig. 1:**
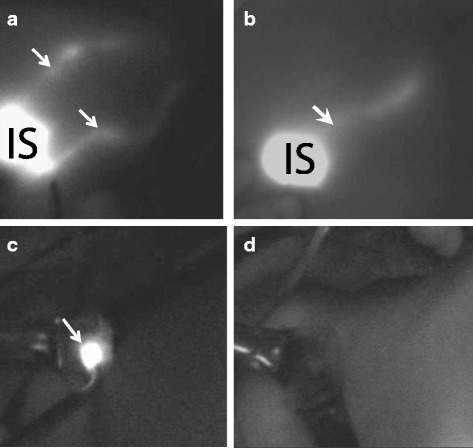
SLN resection mapping by ICG fluorescence. **a** Two streams of dermal lymphatic vessels. **b** One stream of dermal lymphatic vessels (IS—injection site; white arrow—fluorescent lymph vessels). **c** SLN highlighted by ICG fluorescence (white arrow). **d** Fluorescence disappeared after all the SLNs were removed

Patient and tumor characteristics are shown in Table 1. Transcutaneous fluorescent lymphography was visible in 184 breasts. A routine incision used in dye-guided SLNB in the armpit was made in the other 16 procedures. When the skin and subdermal fat were incised, fluorescent lymph nodes could be successfully detected in 194 axillas, including 15 cases with no visible fluorescent flow in the skin (Fig. 1).

**Table 1:** Patient and tumor characteristics

Characteristics	*N*	%
Age		
52 (33–74)		
≥ 50	112	56.5
< 50	86	43.4
T		
T1	138	69
T2	61	30.5
T3	1	0.5
Histological type		
In situ	23	11.5
Microinvasive	12	6
Invasive ductal	134	77
Invasive lobular	15	7.5
Other	16	8
Tumor grade		
I	22	11
II	112	56
III	28	14
NA	38	19
Molecular subtype (immunohistochemical)		
Luminal A	65	32.5
Luminal B	78	39
Her-2 positive	9	4.5
Triple negative	44	22
NA	4	2
Laterality		
Left	105	52.5
Right	95	47.5
Quadrant		
Upper outer	94	47
Lower outer	26	13
Upper inner	55	27.5
Lower inner	17	8.5
Central	8	4
Body mass index (BMI)		
< 19	10	5
19–25	120	60.6
≥ 25	68	34.3
≥ 30	0	0

### Nodal identification performance

The detection rates of the tracer agents are shown in Table 2. The overall SLN detection rates for the ICG and dual methods were 97 and 99.5%, respectively, while the detection rate for dye alone was 89% (*χ* ^2^ = 26.2, *p* < 0.001). The difference between the ICG + MB tracing modality and MB alone was therefore 10.5%. The 95% confidence interval (CI) for this difference was 6.1 to 14.9%. Since this CI does not include the superiority margin of 5%, we are powered to reject the null hypothesis and conclude that the combination of ICG and MB is superior to MB alone.

**Table 2:** Detection rates of various tracers

	*N*	Detection rate (%)
Fluorescent+/blue+	199	99.5
Blue+	178	89.0
Fluorescent+	194	97.0

An age ≥ 50, a BMI ≥ 25, an outer upper quadrant tumor and a larger tumor size were associated with a reduced detection rate for the MB alone method but not for the ICG or combination method.

A total of 616 SLNs were detected by one or both methods. The median numbers of SLNs detected by the ICG (fluorescent−/blue−), MB alone (fluorescent−/blue+), and the combination methods (fluorescent+/blue+) were 3 (range 1–8), 2 (range 1–6), and 3 (range 1–8), respectively. The median numbers of SLNs identified by the ICG and combination methods were significantly higher than those identified by MB alone (*p* < 0.001).

Fifty-eight nodes out of 616 SLNs contained metastases. Twenty nodes were fluorescent, 4 were blue, and 34 were double-positive (Table 3), and the numbers of axillary nodes diagnosed by each modality were 10, 2, and 32, respectively. Hence, 22.7% positive axillas would have been missed if the dye only method had been the sole method used in this cohort.

**Table 3:** Positive SLNs and patients detected by different methods

		Detected positive nodes	Detected positive axillas(patients)
		Number (%)	Number (%)
Fluorescent+	Blue+	34 (58.6)	32 (72.7)
Fluorescent+	Blue−	20 (34.5)	10 (22.7)
Fluorescent−	Blue+	4 (6.9)	2 (4.6)
Total		58 (100)	44 (100)

### Adverse effects

No acute or chronic allergic reactions were observed in this study. A total of 148 patients reported colored urine, most of which disappeared in 24 h. Twenty-three patients (23/82) who received breast-conserving therapy reported temporary skin staining, which was a complication of both ICG and MB, and only five patients had permanent tattooing (we defined “permanent” as skin pigmentation ≥ 6 months), which was due to the injection of MB. Although palpable subcutaneous nodules, probably due to fat necrosis at the injection sites, were reported in nine patients, no patient needed a diagnostic biopsy, and there were no reports of skin necrosis.

## Discussion

This was a prospective study to assess whether the addition of the promising ICG method to the widely used MB dye method improved the SLN identification performance in patients with early breast cancer. We conclude that the detection rate of the fluorescence and dye combination was significantly higher than that of the MB dye alone (99.5 vs. 89%). This result is consistent with previous studies [21, 22].

The high identification rate and accurate diagnosis performance of the ICG + MB modality provide the possibility of a non-radioactive alternative method for dual tracing of SLNs. It would be fairly attractive to abandon the use of radioactive agents, especially in institutes where radioisotopes are not readily available. Ballardini B. from the European Institute of Oncology reported a trial comparing ICG with a ^99m^Tc-labeled radiotracer in 134 patients. This equivalently designed study was powered to demonstrate that the ICG method yielded a high detection rate of SLNs that was not inferior to that of radiotracers [23]. Although Ahemd M. subsequently criticized the absence of a standard dual mapping technique in Ballardini B’s study [24], replacing the radiotracer with ICG showed promise. A recent meta-analysis performed by Sugie T. [25] confirmed this assumption. Since the current study was designed as a superiority trial with *δ* set at 5%, we can draw the conclusion from the positive results that the combined use of ICG and MB is more effective than dye alone, as we observed an increase in the detection rate by 10.5%, or in other words, a 5% improvement, which is comparable to the difference between standard radioisotope included method and dye alone [26]. Although there were few studies that compared the identification performance of ICG with standard radiotracer and dye combination method directly, the combined use of ICG and dye is deemed to be the most likely alternative to the current standard.

In addition to the detection performance improvement, ICG may bring accuracy benefits. First and foremost, the extreme sensitivity of ICG contributed to the harvest of extra SLNs that were undetected by the dye. In the current study, the median number of the SLNs detected by ICG + MB was 3, which was greater than that of SLNs detected by MB alone. Similar results were also found in previous studies [18, 21, 27, 28]. Results of two meta-analyses revealed that acceptably more SLNs (three to four nodes) were related to a higher detection rate and a more accurate prognosis, whereas only one SLN was found to not adequately represent the axillary status [29, 30]. Since ICG detected more SLNs, we speculated that the higher sensitivity of ICG was due to its higher visibility via the high-resolution near-infrared equipment in contrast with the perception of dye uptake via the naked eye and that this higher visibility might improve the detection rate of positive nodes. As a consequence, identification of additional positive SLNs missed by the dye method may elevate the accuracy of the biopsy procedure. The present trial demonstrated that adding ICG to MB tracing might contribute to avoiding a false-negative assessment of the axillary status in 22.7% of patients. However, the potential benefits should be confirmed by randomized trials.

As reported before, ICG-guided SLNB had several limitations. For example, the tissue penetration capacity of NIR fluorescence is lower than that of gamma rays. One concern is that the limitation of penetration distance of ICG based tracing would result in worse performance in obese patients. Though no axillary skin compression technique used in the current study which was reported by Kitai [31], we found no significant difference between obese patients (BMI ≥ 25) and non-obese patients. According to Grischke’s research, there was no difference in the detection rate when a cut-off of BMI ≥ 30, or ≥ 35 were used, whereas detection with ICG was only difficult in patients when BMI > 40 [32]. As shown by our study, patients with very high BMI were rare in China. Hence, this limitation would not be an obstacle to the clinical use of ICG. Fluorescence quenching effect was another point regarding the efficacy of ICG navigation. It was reported that the ICG exhibited intense quenching (i.e., reduction of fluorescence emission) as its concentration was increased. Based on their results of a small cohort, Mieog recommended an injection dose of 0.62 mg as an optimal dose for ICG [33]. However, several studies that reported varied doses (between 0·625 and 15 mg in a volume of between 1 and 5 ml) yielded similarly high detection rates [14]. There was no consensus on the optimal dose of ICG. Based on the lymphatic visibility, we chose 1.25 mg in 1 ml as our injection dose. The excellent navigation performance indicated that this dose was clinically acceptable. Adverse effects such as sensitive reaction and skin lesion are the primary concerns regarding the safety of dye-based mapping [34, 35]. In this cohort, we found no allergic cases. Interstitial use in lymph mapping rather than intravenous injection may be the main cause of the fewer instances of allergic reaction. Moreover, absence of an allergic reaction may be due to the exclusion of iodine hypersensitive patients. The main dermal complications included temporary skin staining, permanent tattooing, and subcutaneous nodules at the injection sites. Although these skin complications may lead to certain degrees of anxiety in stressed patients, these complications would be acceptable to most patients. The relatively good safety results were similar to results from previous meta-analyses [14, 19].

The current study had two shortcomings. First, we did not compare the ICG + MB method to the ^99m^Tc + MB method. However, the excellent performance of this dual mapping method might be indirectly confirmed by a superiority test at 5%. Furthermore, regardless of the benefits of self-controlled design, including elimination of individual variation, decreased sample size, and good statistical efficiency, confounding bias may occur. In this setting, after all fluorescent nodes have been detected, surgeons might tend to end the procedure rather than work so hard to search for more blue nodes. This might result in an underestimation of the efficacy of MB and an overestimation of the performance difference.

In summary, this study reveals a high detection rate of ICG and the superiority of combining ICG fluorescence with blue dye in early breast cancer detection strategies. It is proposed that a combination of ICG fluorescence and blue dye (MB) method can be used in centers where radioactive agents are unavailable. Furthermore, for the excellent performance of this novel dual tracing modality, a better non-radioactive substitute for the combination of radioisotope and blue dye beckons.

## Conclusion

The lymphatic navigation by ICG fluorescence detects SLNs at a high detection rate and improves the mapping performance when added to the dye method. The novel ICG + MB dual tracing modality, without involvement of radioactive isotopes, exhibits great potential as an alternative to traditional standard mapping methods.

## Abbreviations

^99m^Tc: Technetium-99m
BMI: Body mass index
CI: Confidence interval
ICG: Indocyanine green
MB: Methylene blue
NIR: Near-infrared
SLN: Sentinel lymph node
SLNB: Sentinel lymph node biopsy
